# Uncertainties in Measuring Soil Moisture Content with Actively Heated Fiber-Optic Distributed Temperature Sensing

**DOI:** 10.3390/s21113723

**Published:** 2021-05-27

**Authors:** Robert Wu, Pierrick Lamontagne-Hallé, Jeffrey M. McKenzie

**Affiliations:** Department of Earth and Planetary Sciences, McGill University, Montreal, QC H3A 0E8, Canada; pierrick.lamontagne-halle@mail.mcgill.ca (P.L.-H.); jeffrey.mckenzie@mcgill.ca (J.M.M.)

**Keywords:** distributed temperature sensing, fiber optic, FO-DTS, soil moisture, hydrology

## Abstract

Actively heated fiber-optic distributed temperature sensing (aFO-DTS) measures soil moisture content at sub-meter intervals across kilometres of fiber-optic cable. The technology has great potential for environmental monitoring but calibration at field scales with variable soil conditions is challenging. To better understand and quantify the errors associated with aFO-DTS soil moisture measurements, we use a parametric numerical modeling approach to evaluate different error factors for uniform soil. A thermo-hydrogeologic, unsaturated numerical model is used to simulate a 0.01 m by 0.01 m two-dimensional domain, including soil and a fiber-optic cable. Results from the model are compared to soil moisture values calculated using the commonly used T_cum_ calibration method for aFO-DTS. The model is found to have high accuracy between measured and observed saturations for static hydrologic conditions but shows discrepancies for more realistic settings with active recharge. We evaluate the performance of aFO-DTS soil moisture calculations for various scenarios, including varying recharge duration and heterogeneous soils. The aFO-DTS accuracy decreases as the variability in soil properties and intensity of recharge events increases. Further, we show that the burial of the fiber-optic cable within soil may adversely affect calculated results. The results demonstrate the need for careful selection of calibration data for this emerging method of measuring soil moisture content.

## 1. Introduction

For environmental monitoring and sensing, soil moisture content is a critical component of the hydrologic system. In near-surface environments, the ground is a combination of soil, water, and air, and is referred to as the vadose zone. Soil moisture is the amount of water within a given soil and changes spatially and temporally [1]. The movement and storage of water in the vadose zone is important for numerous processes, including agricultural engineering, groundwater recharge, predicting the response of streams and rivers to large rainfall events or snowmelt, and geoengineering [2,3,4,5,6]. Soil moisture content is most often measured in situ using sensors such as electrical conductivity or capacitance probes [7]. With proper calibration, these methods can provide very accurate point measurements of soil saturation. However, these sensors are limited in that they provide soil moisture measurements at discrete points and may not capture the intrinsic spatial variability of the subsurface [1]. There have been recent advances in high-resolution remote sensing of soil saturation, but the measurements are limited to very shallow depths [8]. Actively heated fiber-optic distributed temperature sensing (aFO-DTS), the focus of this research, is an emerging technology that has great potential for the measurement of distributed soil moisture [9].

Fiber-optic distributed temperature sensing (FO-DTS) has been utilized in environmental and hydrologic sciences to measure temperature and heat fluxes in rivers, streams, and subsurface boreholes [10]. FO-DTS measures temperature along a fiber-optic cable via nonelastic Brillouin and Raman backscattering. In Raman backscattering, there is a change in the intensity of reflected light when incident light strikes the fiber-optic glass wall and is reflected [10]. The intensity of backscatter at the anti-Stokes frequency is dependent on the temperature of the cable where the reflection occurs, therefore temperature at the point of reflection can be calculated from the ratio of the intensity at the anti-Stokes frequency to that at the Stokes frequency [11,12]. By measuring the two-way travel time of light along the fiber-optic cable, temperature can be measured at sub-metre spacing over kilometers of cable [13]. The precision of this measurement is proportional to the square root of the integration period or the square root of the time in one step interval assuming no errors due to temperature drift [10,12,14]. FO-DTS has been used extensively for temperature monitoring and fire identification [15,16]. FO-DTS systems are now field-portable, durable, compact, and are used for many environmental and hydrologic applications, e.g., [10,17,18].

The aFO-DTS method to measure soil moisture content uses FO-DTS to measure the thermal response of a section of the fiber-optic cable to a controlled heat pulse [14]. In the vadose zone, soil, water, and air have distinct thermal conductivities and heat capacities. If a fiber-optic cable is buried in soil and a heat pulse is created by applying a known electrical current across the steel outer core of the cable, the resulting thermal response (measured with the FO-DTS system) will be a function of the proportions of soil particles, water, and air. Assuming the soil does not change, then the thermal response at a given location is controlled by the amount of water present. As described below, with calibration, it is possible to use aFO-DTS to measure soil moisture [9].

With current off-the-shelf field-ruggedized technology available, aFO-DTS technology has a minimum temperature measurement of 0.125 m along a fiber-optic cable buried in soil, with measurements every 1 s. This measurement density and sampling interval is possible for cables up to 10 km in length. The measurement of water content has great potential with aFO-DTS, but calibration across field scale variable soil conditions is difficult [6,9,14,19,20].

In an applied field-scale deployment of aFO-DTS, the measurement of soil saturation is simultaneously observed at all locations along the fiber-optic cable for each heat pulse event. The changes of thermal responses during a heat pulse at all locations is not only a function of variability in soil saturation but also the spatial heterogeneity of the bulk soil properties, recharge distribution and duration, and the effect of heat pulses on the soil, such as the duration of heating. For subsurface hydrology, recharge events may cause rapid increases in soil moisture content as water from precipitation, snowmelt, or irrigation infiltrates the subsurface and flows downward. Thus, the calibration protocol relating the measured thermal response to soil water content must reflect the particularity of each location along the buried cable. This is challenging due to the limited available information on the spatial variability of soil thermal and physical properties in field scale experiments [1].

Dong et al. [21] provide a strategy for measuring soil moisture with just FO-DTS. They use an adaptive particle batch smoothing algorithm in conjunction with a numerical model to assimilate FO-DTS observations of diurnal soil temperature fluctuations to calculate soil moisture content. Their study assesses variability of soil thermal properties at the scale needed for effectively distributed calibration, though the complexity of their approaches limits an easy application in applied settings.

Soil moisture content values are reported in numerous ways, largely as a function of the technical field. Broadly, agronomists and soil scientists often use volumetric or gravimetric water content, while soil mechanists and hydrogeologists use saturation or degree of saturation. Given soil density and porosity, one can convert between metrics. In the analysis presented herein, we use saturation, which is defined as the fraction of soil pore space occupied by water (i.e., a saturation of 1 indicates the pore space is filled with water and there is no air) [22].

For a field-based aFO-DTS protocol, factors, such as bulk density, mineralogy, organic matter content, and initial temperature, contribute to the spatial variability of heat transport in soils [23]. For example, with an increase in soil compaction, porosity decreases, and as a result, particle contact increases [24]. The increase in particle contact is important in mineral soils where grains have a higher thermal conductivity than water and air. Heat will preferentially conduct across the connected mineral grains instead of the more insulating liquid and gas mediums. Soil composition also contributes to the variability of thermal conductivity. To isolate the effect of permeability and recharge on heat transfer, the research described below considers sand of uniform mineralogy and density.

For aFO-DTS, the thermal response of soil to pulse heating can be calculated as a cumulative temperature increase, T_cum_ (s°C) [14]:

(1) $$T_{\mathrm{cum}}=\overset{t_{0}}{\underset{0}{\int }}\Delta Tdt$$

where *t*_0_ (s) is the duration of the heat pulse integration period, and Δ*T* (°C) is the temperature change with respect to ambient conditions. As T_cum_ is used as a token term for thermal conductivity, all factors that affect thermal conductivity are expected to affect T_cum_. In field deployments of aFO-DTS, a calibration protocol relating T_cum_ to soil saturation should ideally account for the spatial variability of soil thermal properties, which is often non-linear. There are several ways to calibrate the relationship for thermal properties to soil saturation for a given soil:

- Model calibration curves based on field samples with different soil water contents. For example, Benítez-Buelga et al. [19] collected undisturbed field samples and measured the thermal properties of the samples under varying soil saturation conditions to create a calibration function. The calibration function was used in a heat transport model to generate another calibration function relating T_cum_ to soil saturation for the specific soil in the study.
- Field generated calibration curves based on soil saturation probe data. For example, Cao et al. [25] generated calibration curves relating the thermal response of an aFO-DTS experiment to soil saturation content measured by soil saturation probes installed next to defined sections of the heated fiber-optic cable.
- Laboratory generated curves based on soil columns. For example, Wu et al. [26] constructed a soil column with integrated fiber-optic cable. The water table in the column was controlled to impose different soil saturation conditions inside the soil column. The calibration curve relating T_cum_ to soil saturation was obtained by fitting a curve to the T_cum_-soil saturation content collocated measurements.

The calibration protocols described above can be challenging to apply when there is large variability in the background soil thermal properties. Variability in thermal conductivity can influence the relationship between T_cum_ and soil saturation, thus affecting the accuracy of the protocol. In natural and heterogeneous environments, it is often impractical to apply these calibration methods given the wide range of conditions and material properties. Observations from different locations are required to cover the range of spatial variability of soil thermal properties and, even when the range of variability is known, there is little literature detailing the potential errors in assigning aFO-DTS measurements to a particular calibration function.

The research objective of our study is to use a thermo-hydrogeologic numerical model to evaluate potential errors in aFO-DTS measurements of soil moisture content. This study is not intended to present a comprehensive model of aFO-DTS but rather aims to identify and test common assumptions in the heat pulse protocol and to analyze the potential errors in soil moisture calculations in field-based experiments. Using an analysis-based approach, we simulate a base scenario model with uniform parameters. We then vary parameters to test the sensitivity of commonly used assumptions, scenarios, and protocols used in field-based aFO-DTS soil saturation studies. Common untested assumptions, such as the effect of ambient soil saturation, amount of recharge, length of active heating, and the distribution of heterogeneities, are tested.

## 2. Methods

### 2.1. Numerical Thermo-Hydrogeologic Model

The numerical model code used in this research is SUTRA, a finite element model developed by the U.S. Geological Survey that simulates saturated-unsaturated groundwater flow with energy transport [27]. SUTRA uses the Richards Equation to simulate unsaturated porewater flow coupled with conductive-advective energy transport. The model includes temperature-dependent fluid density and the effects of soil saturation on the subsurface hydraulic and thermal properties but does not simulate vapor or air flow. See Voss and Provost [27] for a detailed description of SUTRA’s governing equations. The soil saturation is calculated from the modelled pressure at each time step using the van Genuchten function [28].

SUTRA is a public-domain model that is widely used for groundwater research [29], has been well tested with field data [30,31], and extensively used to simulate coastal hydrology [32,33], water resources [34,35], geothermal reservoirs [36,37], and cold regions’ hydrogeology [38].

### 2.2. Modifications Made to the Model

To adapt the model to simulate aFO-DTS, the SUTRA code was modified in two ways. First, SUTRA calculates the subsurface bulk thermal conductivity with a weighted arithmetic mean from the thermal conductivities of constituents of the porous matrix (i.e., soil particles and water), but not the air phase. However, the arithmetic mean is not considered to be the correct estimation of soil bulk thermal conductivity [39] and ignoring the air phase may amplify these inaccuracies [40]. The SUTRA bulk thermal conductivity equation (*K*) was modified to integrate the air phase with a weighted harmonic mean [39]:

(2) $$K=1/\left[\varepsilon \left(S_{L}/K_{L}+\left(1-S_{L}\right)/K_{A}\;\right)+\left(1-\varepsilon \right)/K_{S}\right]$$

where *ε* is porosity, *S_L_* is water saturation, and *K_L_*, *K_A_*, and *K_S_* are the thermal conductivities of water, air phase, and soil particles, respectively.

The SUTRA code was also modified so that the source of energy (active heating) is applied to all the mesh nodes of the steel core of the fiber-optic cable to cumulatively add energy into the domain over a set period. 

### 2.3. Model Setup

#### 2.3.1. Domain and Mesh

The parameters and domain for the model are based on field and laboratory experiments [6,26,41]. For the field experiment, aFO-DTS was used to measure soil moisture in a constructed sand unit. This field experiment included careful measurement of many of the parameters required for calculating soil moisture from aFO-DTS and provides a reasonable starting point for the numerical modeling. In the numerical model, physical properties of the sand and cable are also based on the laboratory experiment.

**Figure 1:**
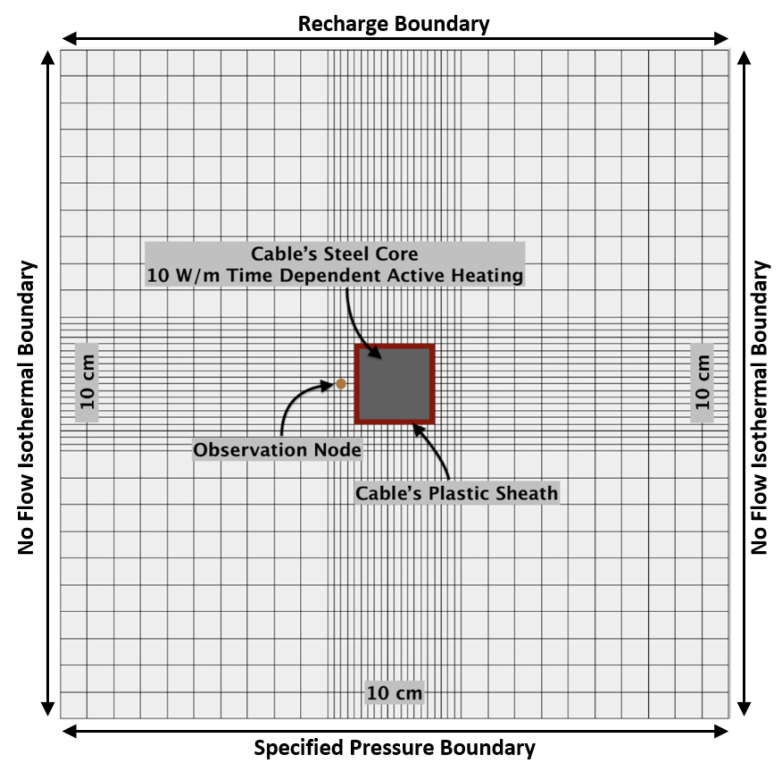
Model domain. The domain is 0.10 m × 0.10 m and contains 0.004 m × 0.004 m elements and 0.001 m × 0.001 m elements in the outer and center areas, respectively. The model domain includes the cable plastic sheath and steel core. Soil moisture is recorded at the observation node.

The model domain is a two-dimensional cross-section containing a simulated fiber-optic cable buried in homogenous, isotropic sand. The base material properties used in the simulations are listed in Table 1. The model’s domain dimension is 0.10 m × 0.10 m. The model mesh layout has 0.004 m × 0.004 m element spacing in the outer bands and 0.001 m × 0.001 m element spacing in the inner bands (Figure 1). Although fiber-optic cables are round, the rectangular representation in the model domain does not affect heat transfer during the simulations as energy is spreading radially at the sand-cable interface.

The model domain represents a homogeneous, medium-grained sand surrounding the fiber-optic cable located in the center of the model domain. The fiber-optic cable is represented by a 0.01 m × 0.01 m steel core surrounded by a 0.001 m thick plastic sheathing. Each medium (sand, plastic, and steel) has its own set of hydraulic and thermal properties (Table 1). The permeability of the steel core and plastic sheath have been set to be effectively impermeable (10^−90^ m^2^) to avoid water flowing through the cable. The representation of the optical fiber is omitted from the simulations as the thermal effect of the thin glass fiber at the center of the steel core is assumed to be negligible.

#### 2.3.2. Boundary Conditions

The vertical sides of the model domain are no-flow boundaries. The top hydraulic boundary condition allows water to flow into the model domain through a time-dependent specified recharge boundary condition (Figure 1). This boundary allows water to enter the domain at specified periods during the model simulations, with varying rates and durations of recharge. The hydraulic boundary condition across the bottom of the domain is a specified pressure boundary condition of −65,000 Pa, which corresponds to the pressure at which residual saturation (0.045) is reached (Figure 1). This boundary condition allows water to exit the model to prevent pooling. The simplicity of this “drain” could induce unintended water flowing upwards from the bottom of the model domain; however, given the model setup, the distance of the drain from the observation area (adjacent to the cable) is sufficiently large that the results would not be affected by such a phenomenon. Water cannot flow out at the top boundary, meaning that all water must be drained at the bottom of the model domain.

The model timestep is one second, with an active heat pulse period of either 15 or 2 min. The energy source is applied to the cable steel with 10 W.

All four model edge boundaries are isothermal, with no heat being conducted into or out of the model. Energy may enter the model through the top boundary via inflowing water during recharge. Along with the heat pulse in the cable’s steel core, this is the only input of heat to the model domain. The temperature of the inflowing water is 20 °C, except when stated. Water being discharged at the bottom model boundary represents the only heat output.

### 2.4. Calibration Curves

To calculate soil moisture with the T_cum_ method, a calibration curve relating cumulative change in temperature to soil moisture conditions is required [20]. We use the numerical model to develop a synthetic calibration curve. Static water conditions, where gravity is set to 0 m/s^2^ and movement of water is negligible, are simulated with the default parameters listed in Table 1. The parameters, in theory, represent the ideal conditions for measuring a calibration curve due to the absence of recharge and groundwater flow. The static water cases are used to obtain T_cum_ values corresponding to each specific soil saturation condition for 10% soil moisture increments, from 10% to 100% saturation. The temperature used to calculate T_cum_ is observed at the center of the steel core where the fiber-optic cable is located. Soil saturation is recorded at a node in the sand that is 0.004 m to the left of the modeled cable (Figure 1). This node can be conceptualized as a point source moisture probe.

The calculation of T_cum_ is obtained from the integration of Δ*T* over the time interval of the heat pulse at the observation node ([14], Equation (1)). Initial temperature conditions are that of 1 s before the start of the heat pulse. Several studies suggest averaging several minutes prior to the start of the heat pulse would produce a more accurate value of ambient temperature. However, Wu et al. [6] noted that this suggestion is impractical for repeated heat pulse cycles because temperature fluctuation following a heat pulse may exceed 1 h, dependent on the soil and heat pulse properties.

The resulting T_cum_ relationship to soil moisture following a heat pulse is nonlinear. There are many suggestions in the literature to calculate soil saturation from a heat pulse based on the specific experimental design and soil properties. For the purpose of our default scenario, we find that a cubic function fitted by the least squares method is the best method to calculate soil saturation from T_cum_.

To compare the accuracy of the static calibration curve, two additional calibration curves are considered for scenarios with recharge, and are generated from simulations following the same conditions, with three exceptions:

1. The initial pressure of these simulations is set to −65,000 Pa.
2. Gravity is set to 9.81 m/s^2^ to allow vertical flow.
3. The top hydraulic boundary condition was changed to a constant specified recharge for 15 min or 2 min heat pulses. As described below, the recharge rates were calculated to provide the model with enough water to reach the total saturation levels tested previously in the static simulations (10% increments).

### 2.5. Protocol Evaluation

The performance of the aFO-DTS protocol to calculate soil moisture content is evaluated in comparison to the observation node with respect to soil saturation and time. In the results and figures, the Saturation Offset is the difference between the aFO-DTS calculated results and that of the model simulations recorded at the observation node. The Real Saturation is the saturation measured by the observation node for a given simulation, unless otherwise indicated.

The Nash–Sutcliffe Efficiency (NSE; [42]) and the coefficient of determination (R^2^) are used as performance metrics. The NSE is a useful metric in assessing the quality of time series in hydrological models by analyzing the protocol’s ability to predict along a 1:1 comparison line. The NSE is calculated as [42]:

(3) $$\mathrm{NSE}=1-\frac{\sum {\left(\theta_{obs}-\theta_{calc}\right)}^{2}}{\sum {\left(\theta_{calc}-{\bar{\theta }}_{obs}\right)}^{2}}$$

where $\theta_{obs}$ the modelled observation node soil saturation, $\theta_{calc}$ the protocol calculated soil saturation from T_cum_, and ${\bar{\theta }}_{obs}$ the mean of the soil saturation time series from the observation node. A value of 1 indicates no variance across the 1:1 line of the time series and that the protocol is perfectly reproducing modelled soil saturation at the sand interface. Conversely, a value of 0 suggests the variance in the time series is equal to the variance of the model.

### 2.6. Model Scenarios

Model scenarios with different conditions and calibration curves were used to evaluate how these parameters would affect model sensitivity (Table 2).

#### 2.6.1. Static Water with 10% Saturation Increments to Build Calibration Curve

The default model parameterization (Table 1) is used to build the initial calibration curve, which represents the relationship between T_cum_ and soil saturation for static water conditions. Gravity is 0 m/s^2^, pressure is held constant, and all sides of the model are set as no flow boundaries to prevent flow. In these scenarios, the hydraulic pressure in the model domain is homogeneous, constant, and set to its respective soil saturation content as calculated from the van Genuchten function (ex., −65,000 Pa for a residual saturation of 0.045). The initial temperature is 20 °C everywhere. The default heat pulse duration is 15 min at 10 W/m (the model is 1 m thick). The resultant heat pulse measured in the fiber-optic steel core is calculated from T_cum_, and a calibration curve is derived using the soil saturation at the observation node. Simulations were made from residual to full saturation at 10% increments. The changes and error associated with the protocol calculation are assumed to only change with soil saturation and thermal properties of water in the sand pores.

#### 2.6.2. Static Calibration with Varying Water Temperature and Continuous Heat Pulses

To test the accuracy of the aFO-DTS protocol, different model scenarios with varying input parameters are simulated using the same no-flow (static) conditions described above (Section 2.6.1). The initial temperature in the default scenario is 20 °C. To test the effect of bulk temperatures on protocol performance, initial uniform temperatures of 10 °C and 5 °C are simulated. There are no cases in the literature of using aFO-DTS at or below freezing temperatures as the active nature of the protocol renders measurements in the presence of frozen ground impractical. The heat generated by the fiber-optic cable would both melt pore ice and change the bulk thermal properties of the soil [38].

An aFO-DTS methodological assumption is that the change in temperature during the heat pulse is not affected by the antecedent temperature. Wu et al. (2020) observed that antecedent heat pulse temperatures do not return to ambient conditions following successive succeeding heat pulses. A 24-h test of 15-min heat pulses every hour is used to assess the error associated with this assumption, and this test is simulated for every 10% saturation increment.

#### 2.6.3. 5 mm to 30 mm Recharge Events—15 min Flowing Water Calibration

For field setting in which the aFO-DTS method is calibrated with field sensors, the potential effect of flowing water on the accuracy of calibration has not been previously systematically evaluated. Using the previously measured static water calibration, simulations are used to evaluate how flowing water affects aFO-DTS measurements. Gravity is 9.81 m/s^2^. A new calibration curve replaces the static calibration based on the general heat pulse curve characteristics observed. The saturation calculated by the protocol is now tested against the observation node in the sand adjacent to the cable. The heat pulses are 15 min every hour for 24 h in initially dry (residual soil saturation) conditions, and an NSE is reported for the entire time series. Recharge into the top of the model begins after the first hour, with a one-hour duration. The protocol is tested with cumulative 5 mm recharge increments (from 5 mm to 30 mm recharge).

#### 2.6.4. 5 mm to 30 mm Recharge Events—2 min Flowing Water Calibration

To test if the duration of the heat pulse affects accuracy, a shorter, 2 min heat pulse period is compared with the previous 15-min integration period using the same recharge values and parameters of the previous simulation.

#### 2.6.5. 5 mm to 30 mm Recharge Events—2 min, 20 mm/hr Flowing Water Calibration

We evaluate developing a calibration using values from the 20 mm recharge test and compare it to the accuracy from the 15 min and 2 min initial calibrations. The purpose of these simulations is to evaluate if an increase in measurement accuracy is obtained when the calibration curve is measured for a specific recharge rate. The recharge rate of 20 mm/hr is chosen because it is at the midpoint between the lowest and highest rates tested, 5 mm/hr and 30 mm/hr, respectively. All other parameters remain the same as the 2 min and 15 min calibration simulations.

#### 2.6.6. Varying Recharge Duration and Soil Heterogeneity

With the 2 min, 20 mm/hr flowing water calibration curve (Section 2.6.5), the accuracy associated with varying recharge duration is evaluated for events of 20 min, 40 min, 80 min, and 100 min. The purpose of these simulations is to measure the accuracy of the protocol using a specified recharge rate calibration when the recharge length is not 1 h. While the recharge rate remains the same, the total amount of water, and thus the velocity of the wetting front, is different from the one-hour tests.

#### 2.6.7. Soil Heterogeneity

The effect of heterogeneity in the soil matrix is evaluated using the Section 2.6.5 calibration with different scenarios. First, the sand domain’s permeability is adjusted to a gaussian distribution with extremums of two orders of magnitude below and above the default permeability value of 10^−12^ m^2^. To reduce the impact of local heterogeneity at the point of measurement, the soil saturation measurements are compared at three observation nodes, located above, to the left, and to the right of the cable. The left observation node is shown in Figure 1, and the above and right observation nodes are also 0.002 m from the edge of the cable.

Macropores are large, vertical openings in the soil, effectively acting as pipes to quickly route water through the subsurface [43]. A vertical macropore is added to the domain with a permeability value at 2, 4, and 6 orders higher than the default sand. Additionally, scenarios in which the permeability of the same area is decreased three orders of magnitude are also tested to represent a potentially compacted layer surrounding the cable. The porosity for the macropore is 0.90. The observation node stays within the sand layer at default parameters, outside of the macropore.

During burial of a fiber-optic cable, the physical soil texture is altered and the permeability around the cable is different from the surrounding conditions. To reduce soil disturbance effects, aFO-DTS data acquisition is often initiated weeks after cable burial to allow time for the soil structure to return to its original state. In some cases, vibratory presses are used to accelerate this process [9]. Nevertheless, soil porosity and permeability structure may be altered with the installation of the fiber-optic cable. A lower permeability around the cable is possible following compaction, or the inverse may occur without subsequent compaction. Further, repeated heating cycles can also cause a change in the contact between the cable and soil, and thus the permeability around the fiber-optic cable [24]. To test the effect of compaction around the cable, we simulate a 0.0002 m thick zone surrounding the cable with a permeability of 2, 4, and 6 orders of magnitudes lower and higher than the default value of 10^−12^ m^2^.

Natural soils have different layers or horizons, with different physical properties. Three additional scenarios evaluate how a low permeability layer would affect the aFO-TDS results. These scenarios are simulated with a 0.01 m thick horizontal low-permeability layer located 0.01 m above the cable, continuous across the entire model domain (i.e., from the left vertical boundary to the right vertical boundary). Three cases are evaluated with a small 0.001 m wide opening with the default permeability, located 0.02 m to either side of the cable and directly above the cable. The low permeability zone (10^−14^ m^2^) is two orders of magnitude lower than the default value, 10^−12^ m^2^.

## 3. Results and Discussion

### 3.1. Static Simulations

The static water cases are set at predetermined soil saturation levels, with water remaining stationary throughout the simulations (see Methodology and Table 2). Using a cubic function to calculate the T_cum_ to soil saturation calibration curve yields an R^2^ of 0.99 between the simulated and calculated soil moisture values.

**Figure 2:**
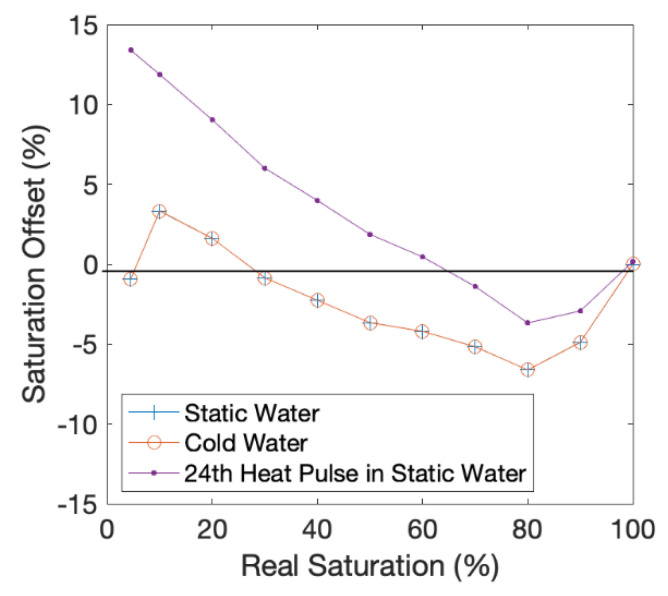
Static Simulations: The plot displays the effects of cold-water infiltration, and the cumulative effect of repeated hourly heat pulses on the accuracy of the protocol calculation. Real saturation refers to the saturation that is prescribed with the model setup.

The effect on accuracy of the aFO-DTS protocol of water colder than the antecedent ambient temperature of the bulk medium is negligible (Figure 2). The drop in water temperature to 10 °C and 5 °C from an initial 20 °C produced a change in soil saturation value of ±0.01%, and the R^2^ remains 0.99.

**Figure 3:**
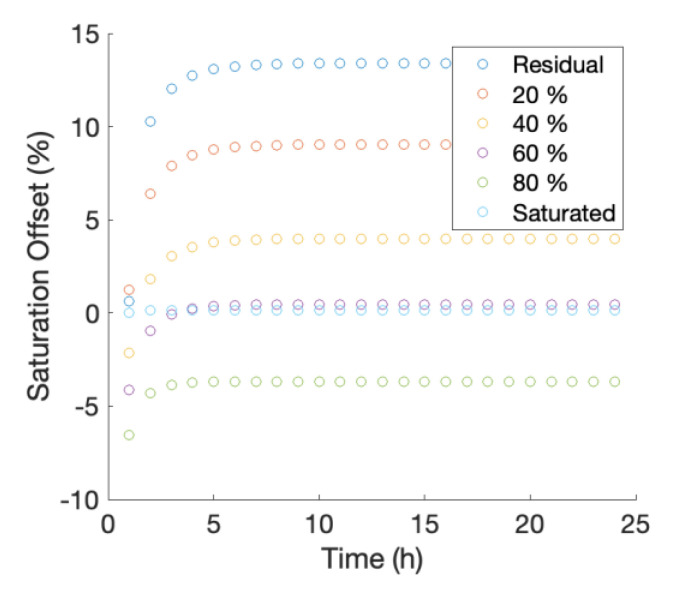
Effect of repeated heat pulses (one cycle per hour, for 24 h) on aFO-DTS accuracy for different saturations. Accuracy, represented by saturation-offset, decreases over the first four heat cycles after which the offset is constant for subsequent heat pulses.

The effect of 24 cycles of 15 min heat pulses at one-hour intervals results in an R^2^ of 0.99 when comparing the soil saturation at the observation node to the calculated protocol value at the end of the 24th cycle. The saturation offset (i.e., the difference in aFO-DTS calculated and observation node value) is within ±4% for saturations at or above 40% (Figure 3). Below 40% saturation, the aFO-DTS protocol has a saturation bias greater than +4%, and the offset is 13.4% at residual saturation levels. This suggests that the error associated with repeated heating cycles at low saturation may be a potential source of error. The error in the saturation calculation initially increases with each heat pulse but reaches a plateau by the 4th cycle. A shorter integration period may reduce the offset at lower saturations.

### 3.2. Simulations with 15 Min Calibration and Recharge

**Figure 4:**
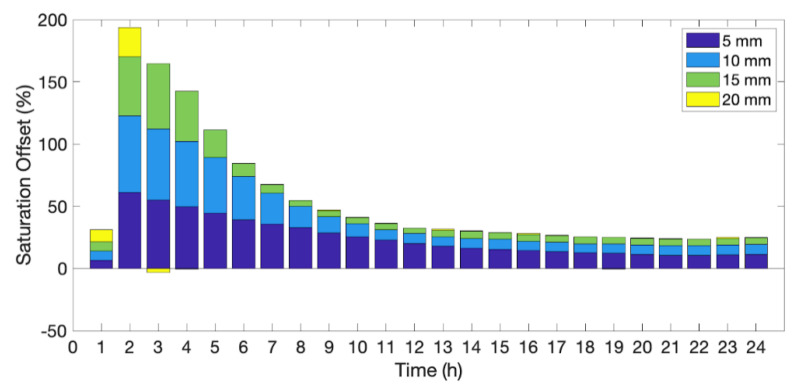
aFO-DTS results using the 15-min calibration for 5, 10, 15, and 20 mm of recharge. Recharge occurs during the second hour of the test. The bar chart is cumulative; for example, at 2 h, the saturation offset for the 20 mm simulation is 39%.

Simulations have set vertical recharge rates. Simulated recharge rates vary from 5 mm/hr to 30 mm/hr to understand aFO-DTS errors across a broad spectrum of recharge intensities (Figure 4). The recharge period is one hour and starts at the beginning of hour two of the simulation. The saturation offset is calculated as the difference between these simulations to that of the 15-min static calibration curve (see Section 2.6.1).

The resulting saturation offset is much larger than for the static calibration cases. The NSE is 0.57 for 30 mm recharge, and 0.07 for 5 mm. The highest offset is during the second hour of the test when recharge is actively being applied to the model domain. The offset is 59% and 39% for 5 mm/hr and 20 mm/hr of recharge, respectively. By the fourth hour, the offset in the 20 mm/hr test is reduced to below 12% and the offset in the 5 mm/hr test is below 28%.

The large offset is a result of the calibration method. T_cum_ is measured during a 15-min period, during which time it is assumed the saturation levels remain constant. With the introduction of flowing pore water, saturation changes throughout the heat pulse measurement period. Heat transfer not only increases through the wetting front, but additional cooler recharge water following the wetting front removes additional heat which is not experienced with the static simulations. The calibration curve is also more sensitive to the length of the heat pulse at lower water contents due to its shape where smaller differences in T_cum_ at lower saturations account for larger changes in soil saturation. For example, the difference in T_cum_ between 10% and 20% saturation is 614 s°C and between 90% and 100% saturation is 1269 s°C.

### 3.3. Simulations with 2 Min Calibrations

**Figure 5:**
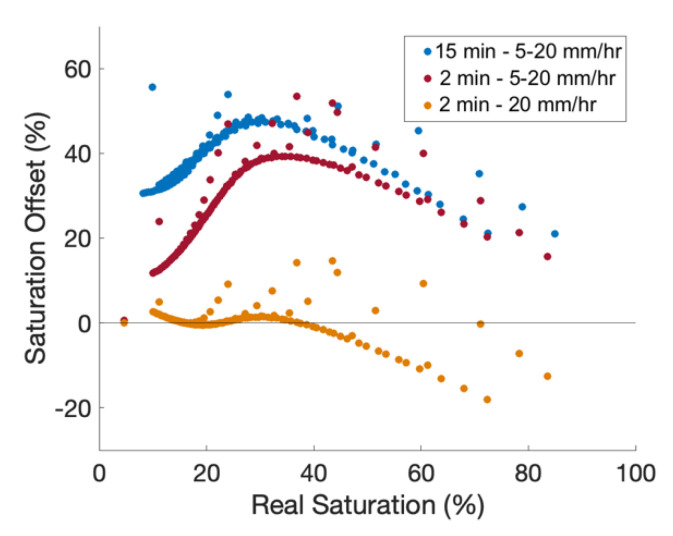
Comparison of three calibration curves: a 15 min heat pulses with recharge rates of 5 to 30 mm/hr, a 2 min heat pulses with 5 to 30 mm/hr of recharge, and a 2 min heat pulses with 20 mm/hr recharge. The first and second calibrations incorporate a range of recharge values (i.e., 5 to 30 mm/hr) in the training set, while the third uses only the 20 m mm/hr recharge data. All three calibrations methods are tested using a full range of recharge scenarios from 5 to 30 mm/hr. Real Saturation refers to measurements at the observation node.

To improve the accuracy of the aFO-DTS calibration, a shorter integration period is evaluated. The results marginally improve from the 15-min tests (Figure 5). Using a 2-min heat pulse resulted in a maximum offset of 41% for a 5 mm recharge period, a decrease of 18% compared to the corresponding 15-min calibration case. The NSE is 0.63 for 30 mm recharge, and 0.57 for 5 mm. To account for the effect of flowing water removing excessive heat, a new calibration curve was arbitrarily made with a 20 mm/hr recharge event for the 2-min flow calibration. The calibration curve has a relationship and results in an NSE of 0.99.

**Figure 6:**
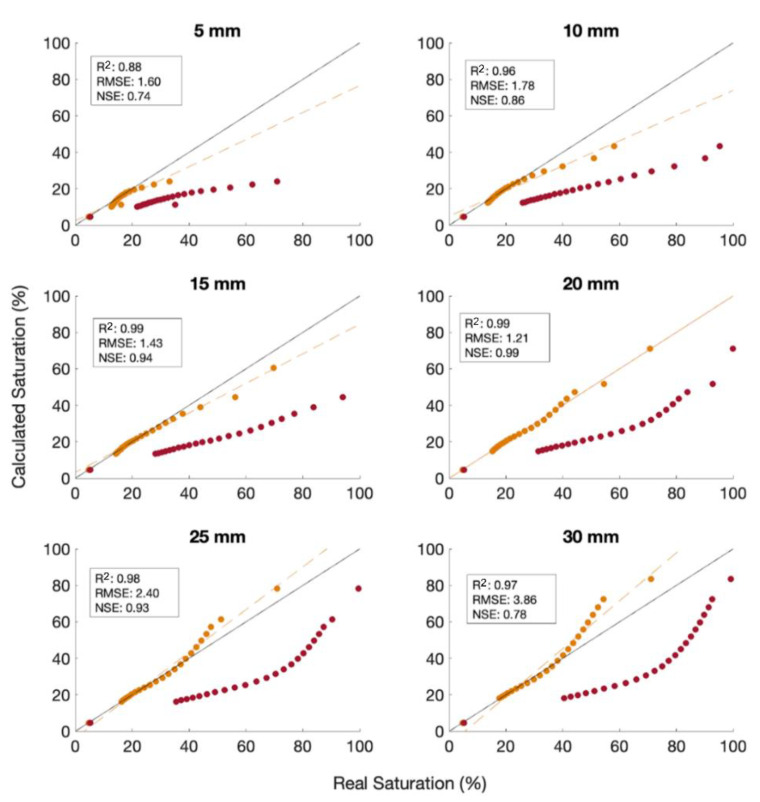
Simulation results using the 2 min-20 mm/hr calibration with recharge from 5 mm/hr to 30 mm/hr (orange). The results using the 2-min flow calibration (red) are displayed as reference.

There is better agreement at all recharge rates using the 2 min 20 mm/hr calibration curve than for the 2 min calibration, with the lowest agreement having an NSE of 0.74 at 5 mm/hr (Figure 6). We interpret these results to indicate that for a field test, specific calibration curves should be measured for the range of expected recharge. The accuracy of the calibration can decrease if the range of recharge rates is high. In our model, a 50% change in recharge rate can cause the accuracy of the calibration to be offset by 25%. Further, the calibration accuracy is lower at higher saturation, i.e., the aFO-DTS calibration underestimates saturation at lower recharge rates and overestimates at higher recharge rates.

### 3.4. Simulations with Variable Recharge and Heterogeneous Soil Conditions

**Figure 7:**
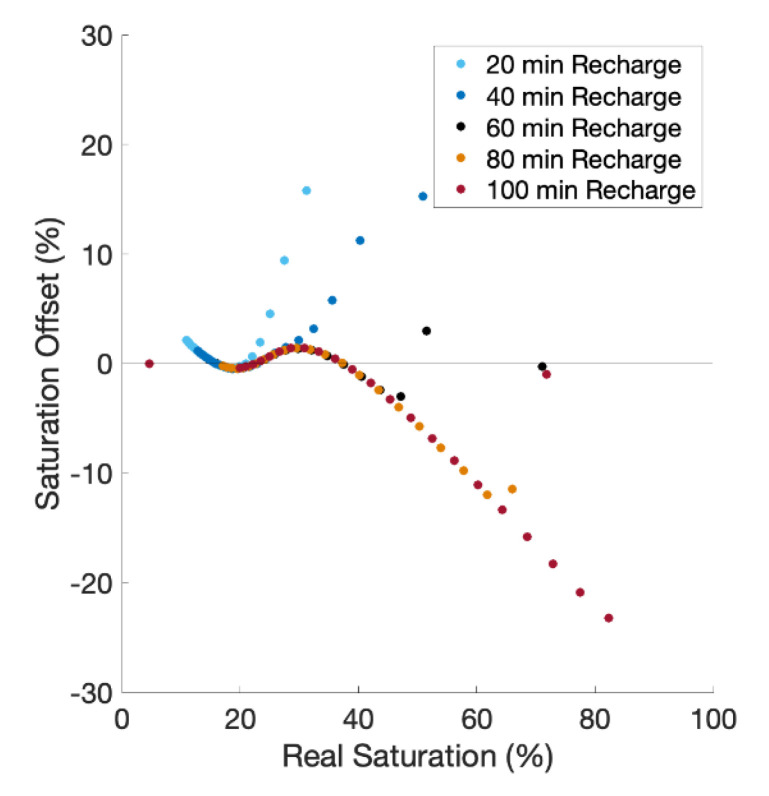
Results for simulations with shorter and longer recharge periods compared to the default 1 hr recharge. All simulations have a constant recharge rate of 20 mm/hr.

The 2 min-20 mm/hr calibration curve improves saturation offset but may present accuracy challenges for different recharge durations. The length of recharge in the previous tests is one hour and occurs during the second hour of the 24 h simulation. We examine the impact of time duration of the recharge event and find that the accuracy decreases when the recharge duration is not one hour. By lowering the recharge time to 40 min and to 20 min, the NSE is 0.90 and 0.79, respectively (Figure 7). Similarly, when increasing the recharge duration to 80 and 100 min, the NSE becomes 0.91 and 0.67 (Figure 7). The calibration tends to overestimate saturation at lower recharge time lengths and underestimate at higher durations. This phenomenon may be due to the velocity of the watering front. Shorter or longer recharge events will lead to different front velocities than the default one-hour duration, resulting in changes to the rate at which flowing water is removing heat from the medium surrounding the cable.

### 3.5. Simulations with Spatially Variability of Permeability

Simulations with spatially variable permeability were used to test the effect of heterogeneity. The spatial distribution of permeability heterogeneity follows a gaussian distribution of two orders of magnitude around the default permeability value. Three observation nodes are placed to the left, right, and top of the cable 0.02 m away from the outer edge of the cable. The aFO-DTS method overestimated the saturation compared to that measured by the observation node in all cases. However, the NSE is 0.99 compared to the average areal saturation of the sand from the three observation nodes. This suggests that calibration and validation with *in-situ* probes in heterogeneous soils may be problematic if the distribution of the soil is spatially biased, highly heterogeneous, or poorly sorted. A laboratory calibration protocol may be needed in such instances because high variability in testing parameters will need specialized calibration tailored to the soil.

**Figure 8:**
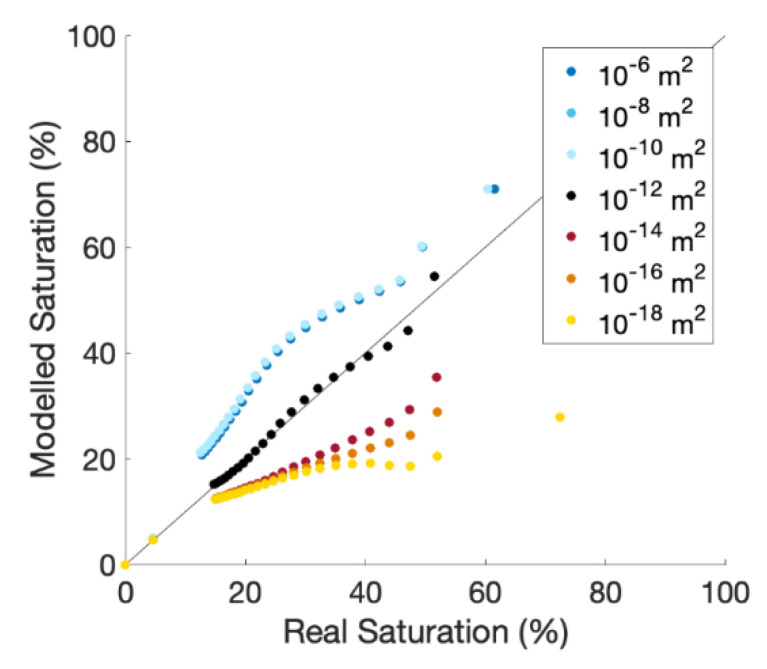
The effect on aFO-DTS accuracy due to permeability values changing in the region surrounding the fiber-optic cable relative to the saturation measured by the observation node with the default permeability conditions. These simulations test the effect of variability in soil permeability rather than the protocol’s ability to account for changes in permeability relative to the observation node’s location.

We evaluated the effect of permeability changes in the region immediately surrounding the cable. The results for higher permeabilities yield an NSE of 0.64, 0.60, and 0.60, respectively (Figure 8). The lower permeability range yielded an NSE of 0.46, 0, and 0. This is an important factor to consider as our model shows that a two orders of magnitude difference in permeability can decrease the accuracy of a protocol by half. Therefore, care should be taken to minimize disturbances to the soil during burial and reduce excessive heating in long-term field tests.

**Figure 9:**
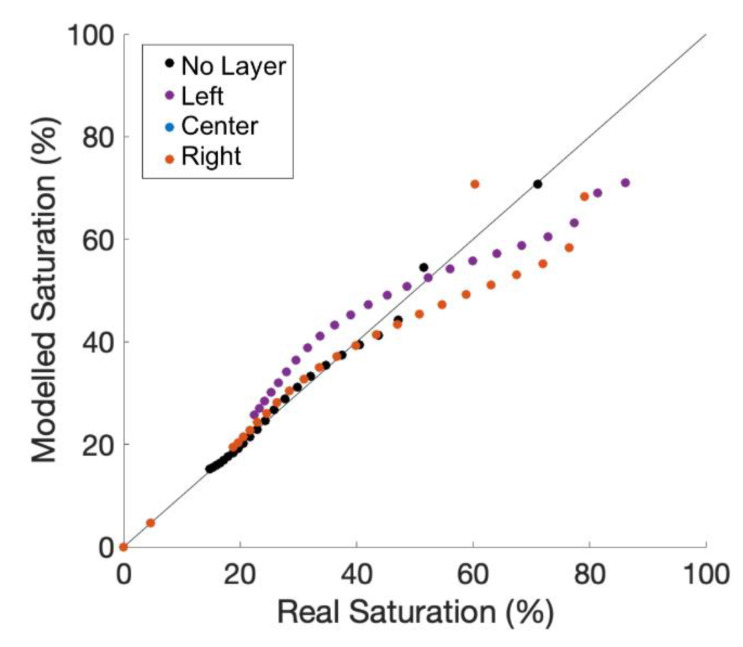
The simulation results of a 0.01 m thick low permeability layer with a small gap to the left, right and directly above the cable. Note, the observation node remains to the left of the cable.

Three additional simulations evaluated the effect of a low permeability horizontal layer above the cable with a gap or “hole” in the layer above, to the left, and to the right of the cable. The NSE is 0.77, 0.75, and 0.46 in the center, left, and right tests, respectively (Figure 9). Note that the observation node is left of the cable in all three tests. The similar NSE values between the above and left cases are expected due to the proximity between the observation node and the cable. The third simulation diverts water on the right side of the model, where the cable is between the observation node and draining water, shielding the observation node from the water resulting in lower accuracy of aFO-DTS calculation during the time-series.

## 4. Conclusions

A numerical model of an aFO-DTS system was developed to evaluate and understand potential errors with this emerging sensor for measuring soil moisture content. Using the model, we evaluate how different calibration approaches, recharge rates, and soil characteristics may affect aFO-DTS results. In summary, our research provides new findings, including an improved understanding of the importance and errors associated with calibration methods for aFO-DTS, an assessment of the limitations of the method due to variable recharge rates, and an understanding of how soil heterogeneity may affect results, including soil disturbance during cable burial.

We find that the calibration method used for aFO-DTS is critical for producing robust results. We employed a simple cubic function to relate model saturation to T_cum_ with a high degree of accuracy. However, when model parameters change, we observe decreases in accuracy. We find that using static water content to develop the calibration curve, such as would be developed in a laboratory setting, may lead to erroneous results in situations with flowing porewater, such as during recharge events. A calibration curve developed for a site-specific soil, preferably measured *in situ*, provides more accurate results. In principle, calibration curves for similar soils should have a similar characteristic shape that is scalable with a few measured T_cum_—soil moisture couplets. Changing the protocol parameters, such as the duration of active heating and the number of heating cycles, decreased the accuracy of the protocol.

Heterogeneity in soils is difficult to account for in field-based aFO-DTS studies. Our results show that variable recharge rates and localized macropores are challenging to account for and can be a potential source of error in aFO-DTS measurements. The soil saturation values were most sensitive to a low permeability layer surrounding the fiber-optic cable, and in some cases would reduce NSE to 0. This case highlights the importance of having adequate contact between the cable and soil and allowing for an appropriate timeframe for soil regeneration following direct cable burial. While disturbances are likely to cause higher permeability adjacent to the cable, lower permeability area adjacent to the fiber-optic cable can be caused by hysteresis in fine-textured soils. Managing the aFO-DTS protocol by lowering the amount and duration of heat generated by repeated active heat pulses may reduce this problem.

Active FO-DTS measurements offer a very powerful tool to measure distributed soil moisture content. Heterogeneity is a fundamental challenge in subsurface hydrology that is difficult to overcome with isolated point measurements. With the ability to deploy kilometres of cable, aFO-DTS can provide unprecedented measurement capability. However, as our analysis shows, care must be taken in evaluating results. The fundamental challenge is that the method requires a valid calibration that may not always be applicable for varying recharge and soil conditions.

## Figures and Tables

**Table 1:** Base model parameters.

Default Parameters	Value
Sand	
Porosity	0.44
Specific Heat of solid (J/kg°C)	840
Thermal Conductivity of solid (J/sm°C)	3.5
Absolute Permeability (m^2^)	10^−12^
Residual Saturation	0.045
Air Entry Value ((kg/(s^2^ m))^−1^)	0.00035
Pore Size Distribution	3.19
Density of solid (kg/m^3^)	2600
Soil Characteristic Curve (van Genuchten Type [27,28])	
Residual Saturation	0.045
Alpha	0.00035
VN	3.19
Cable Plastic Sheath	
Porosity	0.001
Specific Heat (J/kg°C)	1.67
Thermal Conductivity (J/sm°C)	0.026
Permeability (m^2^)	10^−90^
Cable Steel	
Porosity	0.001
Specific Heat (J/kg°C)	502
Thermal Conductivity (J/sm°C)	13.389
Permeability (m^2^)	10^−90^
Energy Source (J/s)	10
Additional SUTRA Parameters	
Water Specific Heat (J/kg°C)	4182
Water Thermal Conductivity (J/sm°C)	0.6
Water Compressibility ((kg/(s^2^ m))^−1^)	4.47 × 10^−10^
Longitudinal and Transverse Dispersity	0.5
Air Thermal Conductivity (J/sm°C)	0.025

**Table 2:** Summary of simulations.

Scenarios	Gravity (m/s^2^)	Initial Temp (°C)	Saturation Comparison	Heat Pulse Duration (min)	Number of Hourly Cycles	Testing Specific Saturation or Time Series	Calibration	Range of Recharge Rate Used (mm/hr)
Static Calibration, Default	0	20	% of Water Specified in Pores	15	1	Saturation (R^2^)	15 min static	0
Static Calibration, Low Initial Temp	0	10, 5	% of Water Specified in Pores	15	1	Saturation (R^2^)	15 min static	0
Static Calibration, 24 Heat Pulses	0	20	Observation Node	15	24	Saturation (R^2^)	15 min static	0
15 min Flowing Water Calibration	9.81	20	Observation Node	15	24	Time Series (NSE)	15 min flow	5–30
2 min Flowing Water Calibration	9.81	20	Observation Node	2	24	Time Series (NSE)	2 min flow	5–30
2 min–20 mm /hr Flowing Water Calibration	9.81	20	Observation Node	2	24	Time Series (NSE)	2 min flow specified for 20 mm/hr	5–30
Permeability Heterogeneity	9.81	20	Observation Node	2	24	Time Series (NSE)	2 min flow specified for 20 mm/hr	5–30
Macropore	9.81	20	Observation Node	2	24	Time Series (NSE)	2 min flow specified for 20 mm/hr	5–30
Permeability Surrounding Cable	9.81	20	Observation Node	2	24	Time Series (NSE)	2 min flow specified for 20 mm/hr	5–30
Overlying Low Permeability Layer	9.81	20	Observation Node	2	24	Time Series (NSE)	2 min flow specified for 20 mm/hr	5–30
Recharge Duration	9.81	20	Observation Node	2	24	Time Series (NSE)	2 min flow specified for 20 mm/hr	20

## Data Availability

The results presented in this study use the public-domain SUTRA numerical code [27].

## References

[1] Brocca L., Melone F., Moramarco T., Morbidelli R. (2010). Spatial-temporal variability of soil moisture and its estimation across scales. Water Resour. Res..

[2] Amin A., Zuecco G., Geris J., Schwendenmann L., McDonnell J.J., Borga M., Penna D. (2020). Depth distribution of soil water sourced by plants at the global scale: A new direct inference approach. Ecohydrology.

[3] Hausner M.B. (2010). Estimating in Situ Integrated Soil Moisture Content Using Fiber-Optic Distributed Temperature Sensing (DTS) Measurements in the Field. Ph.D. Thesis.

[4] Scanlon B.R., Healy R.W., Cook P.G. (2002). Choosing appropriate techniques for quantifying groundwater recharge. Hydrogeol. J..

[5] Tyler S.W., Wheatcraft S.W. (1989). Application of fractal mathematics to soil water retention estimation. Soil Sci. Soc. Am. J..

[6] Wu R., Martin V., McKenzie J.M., Broda S., Bussière B., Selker J., Aubertin M. (2021). Fiber optic measurements of soil moisture in a waste rock pile. Groundwater.

[7] Dorigo W.A., Wagner W., Hohensinn R., Hahn S., Paulik C., Drusch M., Mecklenburg S., van Oevelen P., Robock A., Jackson T. (2011). The international soil moisture network: A data hosting facility for global in situ soil moisture measurements. Hydrol. Earth Syst. Sci..

[8] Wigmore O., Mark B., McKenzie J.M., Baraer M., Lautz L. (2019). Sub-metre mapping of surface soil moisture in proglacial valleys of the tropical andes using a multispectral unmanned aerial vehicle. Remote. Sens. Environ..

[9] Striegl A.M., Loheide S.P. (2012). Heated distributed temperature sensing for field scale soil moisture monitoring. Groundwater.

[10] Selker J., van de Giesen N., Westhoff M., Luxemburg W., Parlange M.B. (2006). Fiber optics opens window on stream dynamics. Geophys. Res. Lett..

[11] Grattan K.T.V., Sun T. (2000). Fiber optic sensor technology: An overview. Sens. Actuators Phys..

[12] Ciocca F., Lunati I., de Giesen N.V., Parlange M.B. (2011). Heated optical fiber for distributed soil-moisture measurements: A lysimeter experiment. Vadose Zone J..

[13] Briggs M.A., Lautz L.K., McKenzie J.M. (2012). A comparison of fibre-optic distributed temperature sensing to traditional methods of evaluating groundwater inflow to streams. Hydrol. Process..

[14] Sayde C., Gregory C., Gil-Rodriguez M., Tufillaro N., Tyler S., van de Giesen N., English M., Cuenca R., Selker J.S. (2010). Feasibility of soil moisture monitoring with heated fiber optics. Water Resour. Res..

[15] Bao Y., Huang Y., Hoehler M.S., Chen G. (2019). Review of fiber optic sensors for structural fire engineering. Sens. Basel. Switz..

[16] e Silva M.S.P., de Barros T.H.C., Alves H.P., do Nascimento J.F., Filho J.F.M. (2021). Evaluation of fiber optic raman scattering distributed temperature sensor between −196 and 400 °C. IEEE Sens. J..

[17] Bense V.F., Read T., Bour O., Borgne T.L., Coleman T., Krause S., Chalari A., Mondanos M., Ciocca F., Selker J.S. (2016). Distributed temperature sensing as a downhole tool in hydrogeology. Water Resour. Res..

[18] Law R., Christoffersen P., Hubbard B., Doyle S.H., Chudley T.R., Schoonman C.M., Bougamont M., des Tombe B., Schilperoort B., Kechavarzi C. (2021). Thermodynamics of a fast-moving greenlandic outlet glacier revealed by fiber-optic distributed temperature sensing. Sci. Adv..

[19] Benítez-Buelga J., Rodríguez-Sinobas L., Calvo R.S., Gil-Rodríguez M., Sayde C., Selker J.S. (2016). Calibration of soil moisture sensing with subsurface heated fiber optics using numerical simulation. Water Resour. Res..

[20] Weiss J.D. (2003). Using fiber optics to detect moisture intrusion into a landfill cap consisting of a vegetative soil barrier. J. Air Waste Manag..

[21] Dong J., Steele-Dunne S.C., Ochsner T.E., van de Giesen N. (2016). Determining soil moisture and soil properties in vegetated areas by assimilating soil temperatures. Water Resour. Res..

[22] Pinder G.F., Celia M.A. (2006). Subsurface Hydrology.

[23] De Vries D.A. (1952). A nonstationary method for determining thermal conductivity of a soil in situ. Soil Sci..

[24] Sourbeer J.J., Loheide S.P. (2016). Obstacles to long-term soil moisture monitoring with heated distributed temperature sensing. Hydrol. Process..

[25] Cao D., Shi B., Loheide S.P., Gong X., Zhu H.-H., Wei G., Yang L. (2018). Investigation of the influence of soil moisture on thermal response tests using active distributed temperature sensing (A–DTS) technology. Energ. Build..

[26] Wu R., Martin V., McKenzie J.M., Broda S., Bussière B., Aubertin M., Kurylyk B.L. (2020). Laboratory scale assessment of a capillary barrier using fibre optic distributed temperature sensing (FO-DTS). Can. Geotech. J..

[27] Voss C.I., Provost A.M. (2002). SUTRA: A Model for 2D or 3D Saturated-Unsaturated, Variable-Density Ground-Water Flow with Solute or Energy Transport.

[28] van Genuchten M.T. (1980). A Closed-form equation for predicting the hydraulic conductivity of unsaturated soils. Soil Sci. Soc. Am. J..

[29] Deeds N.E., Jones T.L. (2011). An Assessment of Modeling Approaches to Brackish Aquifers in Texas.

[30] Bobba A.G. (1993). Field validation of ‘SUTRA’ groundwater flow model to Lambton county, Ontario, Canada. Water Resour. Manag..

[31] Woods J.A., Teubner M.D., Simmons C.T., Narayan K.A. (2003). Numerical error in groundwater flow and solute transport simulation. Water Resour. Res..

[32] Gingerich S.B., Voss C.I. (2005). Three-dimensional variable-density flow simulation of a coastal aquifer in southern Oahu, Hawaii, USA. Hydrogeol. J..

[33] Smith A.J. (2004). Mixed convection and density-dependent seawater circulation in coastal aquifers. Water Resour. Res..

[34] Kurylyk B.L., MacQuarrie K.T.B., Voss C.I. (2014). Climate Change impacts on the temperature and magnitude of groundwater discharge from shallow, unconfined aquifers. Water Resour. Res..

[35] Oki D.S., Engott J.A., Rotzoll K. (2020). Numerical Simulation of Groundwater Availability in Central Moloka‘i, Hawai‘i.

[36] Burns E.R., Williams C.F., Ingebritsen S.E., Voss C.I., Spane F.A., DeAngelo J. (2015). Understanding heat and groundwater flow through continental flood basalt provinces: Insights Gained from alternative models of permeability/depth relationships for the Columbia plateau, USA. Geofluids.

[37] Burns E.R., Bershaw J., Williams C.F., Wells R., Uddenberg M., Scanlon D., Cladouhos T., van Houten B. (2020). Using saline or brackish aquifers as reservoirs for thermal energy storage, with example calculations for direct-use Heating in the Portland Basin, Oregon, USA. Geothermics.

[38] Lamontagne-Hallé P., McKenzie J.M., Kurylyk B.L., Zipper S.C. (2018). Changing groundwater discharge dynamics in permafrost regions. Environ. Res. Lett..

[39] Buntebarth G., Schopper J.R. (1998). Experimental and theoretical investigations on the influence of fluids, solids and interactions between them on thermal properties of porous rocks. Phys. Chem. Earth.

[40] Xie X., Lu Y., Ren T., Horton R. (2020). Thermal conductivity of mineral soils relates linearly to air-filled porosity. Soil Sci. Soc. Am. J..

[41] Dimech A., Chouteau M., Aubertin M., Bussière B., Martin V., Plante B. (2019). Three-dimensional time-lapse geoelectrical monitoring of water infiltration in an experimental mine waste rock pile. Vadose Zone J..

[42] Nash J.E., Sutcliffe J.V. (1970). River flow forecasting through conceptual models part I—A discussion of principles. J. Hydrol..

[43] Beven K., Germann P. (1982). Macropores and water flow in soils. Water Resour. Res..

